# Supplementary material for: Matching sensor ontologies through siamese neural networks without using reference alignment
Source: PeerJ Comput Sci. 2021 Jun 18;7:e602. doi: 10.7717/peerj-cs.602 (PMC8237319; doi:10.7717/peerj-cs.602)
Supplement: Supplemental Information 1 [file peerj-cs-07-602-s001.zip › 304/refalign.html]

# (level 0) Alignment

## Source: http://oaei.ontologymatching.org/2011/benchmarks/101/onto.rdf

## Target: http://oaei.ontologymatching.org/2011/benchmarks/304/onto.rdf

## Correspondences

type = type
:   1.0

howPublished = howPublished
:   1.0

proceedings = inProceedings
:   1.0

volume = volume
:   1.0

annote = annote
:   1.0

PersonList = PersonList
:   1.0

month = month
:   1.0

copyright = copyright
:   1.0

Unpublished = Unpublished
:   1.0

address = location
:   1.0

chapter = chapter
:   1.0

Chapter < InBook
:   1.0

editor = editor
:   1.0

InBook < InBook
:   1.0

Date = Date
:   1.0

series = series
:   1.0

PageRange = PageRange
:   1.0

date = date
:   1.0

title = title
:   1.0

Booklet = Booklet
:   1.0

numberOrVolume = numberOrVolume
:   1.0

LectureNotes = LectureNotes
:   1.0

url = url
:   1.0

MastersThesis = MastersThesis
:   1.0

mrNumber = mrNumber
:   1.0

pages = pages
:   1.0

TechReport = TechReport
:   1.0

startPage = startPage
:   1.0

edition = edition
:   1.0

lccn = lccn
:   1.0

affiliation = affiliation
:   1.0

institution = institution
:   1.0

year = year
:   1.0

isPartOf = isPartOf
:   1.0

organization = organization
:   1.0

publisher = publisher
:   1.0

school = school
:   1.0

Misc = Misc
:   1.0

collection = inCollection
:   1.0

Collection = Collection
:   1.0

isbn = isbn
:   1.0

abstract = abstract
:   1.0

directors = directors
:   1.0

Academic = Academic
:   1.0

location = location
:   1.0

MotionPicture = MotionPicture
:   1.0

Article = Article
:   1.0

Informal = Informal
:   1.0

price = price
:   1.0

Book = Book
:   1.0

book = inMonography
:   1.0

day = day
:   1.0

School = School
:   1.0

PhdThesis = PhdThesis
:   1.0

Proceedings = Proceedings
:   1.0

number = number
:   1.0

Reference = Entry
:   1.0

endPage = endPage
:   1.0

InCollection = InCollection
:   1.0

author = author
:   1.0

note = note
:   1.0

humanCreator = humanCreator
:   1.0

Part = Part
:   1.0

Publisher = Publisher
:   1.0

Manual = Manual
:   1.0

key = key
:   1.0

InProceedings = InProceedings
:   1.0

Journal = Journal
:   1.0

journal = inJournal
:   1.0

Monograph = Monography
:   1.0

keywords = keywords
:   1.0

issn = issn
:   1.0

contents = contents
:   1.0

Institution = Institution
:   1.0

Deliverable = Deliverable
:   1.0

size = size
:   1.0
